# Cytomegalovirus-associated pancytopenia in a four-month-old infant: a case report

**DOI:** 10.1186/s12879-024-10191-9

**Published:** 2024-11-11

**Authors:** Jeevan Gyawali, Sangita Pudasainee-Kapri, Sumit Agrawal, Dhan Bahadur Khatri, Sugat Adhikari, Prem Prasad Dhunagana

**Affiliations:** 1 Internal Medicine Department, Chirayu National Hospital and Medical Institute (CNHMI), Maharajgunj, Kathmandu, 44600 Nepal; 2 School of Nursing-Camden, Rutgers University School of Nursing, Camden, NJ 08102 USA; 3 Pediatric Department, Kanti Children’s Hospital, Maharajgunj, Kathmandu, 44600 Nepal; 4 Pediatric Department, Chirayu National Hospital and Medical Institute (CNHMI), Maharajgunj, Kathmandu, 44600 Nepal; 5 Primary Care Physician, Shreegaun Primary Healthcare Center, Maharajgunj, Dang, 22400 Nepal; 6 Pediatric Department, Patan Academy of Health Sciences – School of Medicine, Lagankhel, Lalitpur, 44700 Nepal

**Keywords:** Cytomegalovirus, Pancytopenia, Infant, Polymerase chain reaction, Valganciclovir

## Abstract

Cytomegalovirus (CMV) is a beta-herpes virus causing common infections, often asymptomatic in healthy individuals. However, it poses increased risks to immunocompromised individuals and can cause congenital infections, leading to severe disabilities. CMV infection can cause significant hematological abnormality. A four-month-old female infant was admitted for decreased feeding for two days. She was severely pale, without hepatosplenomegaly. In initial laboratory investigations hemoglobin, platelet count, and white blood cells were decreased. The patient was transfused with whole blood and referred to a tertiary care center. Further workup, including bone marrow biopsy, showed hypocellular marrow. The Urine CMV Polymerase Chain Reaction (PCR) test returned highly positive with a viral load of 1,700,000 copies/mL. This patient was diagnosed with CMV-associated bone marrow suppression, and she was prescribed valganciclovir at a dosage of 16 mg/kg/dose every 12 h for 6 months. She had shown significant hematologic parameter improvement during subsequent follow-up. Pancytopenia in infancy should include a differential diagnosis for CMV infection. The early recognition and correct infection management, including antiviral therapy and symptomatic treatment, yield a better prognosis.

## Introduction

Human cytomegalovirus (CMV), a DNA virus, is one of eight human herpesviruses. It belongs to the beta-herpesvirus subfamily [1].In nations with higher incomes, the prevalence of CMV has significantly reduced due to improved hygiene and less intimate contact between adults and children. However, in low- and middle-income countries, virtually every adult has had childhood CMV infections. T cells become infected by CMV, which alters their reactions leading to infection [2].

CMV infection is common in healthy children and adults and usually causes no symptoms. However, there are some high-risk categories, such as recipients of immunocompromised organ transplants and those with HIV [3]. In immunocompromised patients, CMV infection can cause significant hematological abnormalities like thrombocytopenia, leukopenia, atypical lymphocytosis, and severe anemia in infancy is rare [4, 5].

The most common intrauterine infection is congenital CMV infection, a leading cause of infectious disability, resulting in hearing loss, vision loss, and neurological issues. Intrauterine transmission of CMV occurs in approximately 1 in 200 to 1 in 30 newborns [6]. Infections in seronegative mothers pose the worst prognosis, though seropositive mothers can also have serious fetal outcomes such as hearing loss, impaired vision, cognitive impairment, and neuromotor deficits [6–8].

Pancytopenia is a decrease in platelets, leukocytes, and erythrocytes [9]. Fanconi anemia, the Shwachman-Diamond syndrome, and congenital dyskeratosis are congenital causes of pancytopenia [10]. Acquired causes of pancytopenia include non-inherited aplastic anemia, infections, poisonings, immunological disorders, destruction of peripheral blood cells, malignant marrow infiltrative disorders, and non-malignant infiltrative disorders [10, 11]. Moreover, pancytopenia is a severe side effect of reactivation of the CMV that needs to be treated immediately, especially in people with impaired immune systems [12].

## Case Presentation

A 4-month-old female infant was brought into Province Hospital in Surkhet, Nepal with a history of decreased feeding for the last two days. She was born at 39 weeks of gestation via cesarean delivery due to a previous cesarean Sect. 5 years ago. She had no history of fever, vomiting, diarrhea, or other symptoms. There were no intra- and post-natal complications.

Examination showed a lethargic infant with a poor tone, but she was not in distress. There was no abdominal distension, organomegaly, or lymphadenopathy. Vital signs were temperature: 36.5 °C, Heart Rate: 140 beats per minute, Respiratory Rate: 28 breaths per minute, Oxygen Saturation: 98% on room air. At the time, hemoglobin, platelet count, and white blood cells were decreased (Table 1). 150 ml of whole blood were immediately transfused in the Province Hospital to the infant due to severe anemia.

**Table 1:** Investigation reports at different times of visit

Parameter	Result (At Primary Center)	Results (At Kanti Children’s Hospital)	Results (Follow-up – 2 weeks)	Results (Followup-6 months)	Reference range:
Hemoglobin	4.5 g/dL	6.5 g/dL,	10 g/dL	12 g/dL	10.5–14.0 g/dL
Platelet count:	10,000 cells/μL	35,000 cells/μL	200,000 cells/μL	2400,000 cells/μL	150,000–450,000 cells/μL
White blood cell	3,000 cells/μL	4,500 cells/μL	7,000 cells/μL	9,500 cells/μL	6,000–17,500 cells/μL

The patient was then referred to Kanti Children's Hospital in Kathmandu, Nepal, one of the tertiary referral centers, for further evaluation and management of the infant. Upon arrival at the Kanti Children's Hospital, repeat laboratory tests were done. The results were similar to those at the primary center (Table 1).

Additional laboratory testing including bone marrow biopsy and aspiration was performed, which showed hypocellular marrow with a marked reduction in hematopoietic cells. Peripheral smear revealed hypochromic, normocytic cells with no atypical cells. Hemoglobin electrophoresis of the parents and infant was performed and was found to be normal, while genetic tests were unremarkable. A hematological consultation was conducted to rule out other pathologies. She was hospitalized for seven days and was given supportive management—fluid maintenance, nutritional support, and antimicrobial treatment for secondary bacterial infections. Further testing was a strongly positive CMV PCR test in the urine, with a viral load of 1,700,000 copies/mL. The patient was diagnosed as having bone marrow suppression associated with CMV infection. A course of oral valganciclovir was initiated at a dosage of 16 mg/kg/dose every 12 h for 6 months.

Subsequent follow-up visits after two weeks of discharge and initiation of valganciclovir showed significant improvement in the infant’s hematologic parameters. The infant was on regular follow-up every two weeks for two months, then monthly for six months, to monitor blood counts, including complete blood count, liver function tests, renal function tests, and the urine CMV viral load. After 6 months of complete dose of antiviral her hematologic parameters were maintained in normal ranges (Table 1). Long-term prognosis will depend on recovery of the bone marrow function and resolution of the CMV infection.

## Discussion

Congenital cytomegalovirus infection is a major public health problem, with an estimated 4,000 to 6,000 affected newborns showing symptoms annually in the United States alone [13]. While generally considered an unrecognized cause of morbidity in neonates, congenital CMV can cause serious illnesses, such as petechiae, hepatitis, pneumonitis, enteritis, nephritis, hemolysis, and bone marrow suppression in infants born small for gestation [14]. Additionally, the CMV infection is linked to severe neurodevelopmental impairments in cognitive and auditory functions. This is especially important in low-resource countries like Nepal, where many infants face delayed detection and treatment due to limited healthcare access, poor public transport, and unequal services in rural areas [15].

The prevalence of congenital CMV infection is not well recognized in Nepal due to limited data, documentation, and research in this area. Health strategies at the national level have not been effectively integrated into programs related to congenital disorders, including CMV infection in infants. Most national health policies have not prioritized such conditions (i.e., CMV infection) as it contributes to significant infant morbidity and thus requires a much-needed public health response of wide scope for congenital CMV infections among the national health priorities of health issues [16]. There is no screening program for CMV infections among infants in Nepal. A recent meta-analysis indicates that the prevalence of congenital CMV infections in lower-middle-income countries (LMICs) was three times higher than in high-income countries due to increased rates of maternal CMV seroprevalence, increased HIV prevalence at the population level, and young age at childbirth [17]. A recent study in Kathmandu Nepal, showed that over 2% of neonates tested positive for CMV, an indication of the magnitude of the problem [18]. However limited evidence is available about the prevalence and burden of congenital CMV infection among infants in Nepal.

The diagnosis of congenital CMV infection is confirmed by demonstrating the presence of infectious virus, viral antigens, or viral DNA in saliva or urine from infected infants [2, 14]. In this case, the quantitative CMV DNA PCR test from a Urine sample was performed. PCR-based assays for the detection of CMV DNA in saliva or urine from neonates are considered the standard diagnostic method to confirm congenital CMV infection [19]. Regular monitoring of CMV viral load via PCR in immunocompromised children allowed for early detection of asymptomatic reactivation.

Anti-viral therapy and symptomatic care have been recommended as the standard of care for the treatment of postnatal CMV infection in infants. Ganciclovir and valganciclovir are recommended antiviral therapy for postnatal CMV infections [20]. Oral valganciclovir (15 to 16 mg/kg/dose every 12 h) is recommended for infants who can take oral therapy. However, if infant is too sick and or unable to take oral medication, then intravenous ganciclovir (6 mg/kg/dose every 12 h) will be a first-line medication that can be transitioned to oral valganciclovir when they are stable and able to take oral therapy [20]. The duration of therapy will depend on three weeks to a few months or longer therapy based on the infant’s clinical condition to resolve CMV-associated viremia and end-organ disease.

The ganciclovir (GCV), a synthetic nucleoside guanine analog, is the most commonly used medication for CMV illness among infants and children [21, 22]. Similar to current treatment recommendations, the 4 months old infant with CMV infection was prescribed oral Valganciclovir 16 mg/kg/dose every 12 h for 6 months [20, 23]. Valganciclovir is a prodrug of ganciclovir that, is quickly converted to ganciclovir in the intestinal wall and liver after being adequately absorbed from the gastrointestinal tract. About 60% of ganciclovir from valganciclovir is bioavailable in its whole. The primary side effects of ganciclovir are thrombocytopenia, fever, diarrhea, anemia, and neutropenia [24]. Valganciclovir exhibits the same toxicity and resistance mutations as ganciclovir. The primary benefit of this medication is its oral dosing capability for valganciclovir, which is enhanced by meal absorption [25].

Infants with virologically confirmed congenital CMV infection should have a complete physical examination, selective laboratory, and cranial imaging studies of the head to determine the severity of the illness and neurological problem. Children at risk for disabilities should have early intervention and proper counseling of parents to achieve the best performance [13]. Infants who are on antiviral medication should be periodically follow-up with providers to monitor for clinical response and virologic response and tested for CBC with differential and platelets count, CMV PCR, and liver function tests.

## Conclusion

In cases of patients presenting with severe anemia, leukopenia, and thrombocytopenia, it is imperative to consider cytomegalovirus (CMV) infection as part of the differential diagnosis. This case report highlights the importance of early diagnosis of pancytopenia linked to CMV infection in infants. Initiating antiviral treatment with IV ganciclovir or oral Valganciclovir, along with supportive care, can greatly enhance clinical outcomes by boosting immune function in a four-month-old infant suffering from CMV infection. It is essential to conduct close and timely follow-ups to assess the patient’s hematologic response to therapy and overall recovery.

This case report has significant implications for the providers and nurses for universal screening of women and infants for CMV infection, and timely diagnosing, treating, and managing infants infected with CMV. In addition, providers and nurses can enhance strategies for preventing CMV infection during pregnancy and or postpartum by educating on personal hygiene practices including hand washing with soap and water, avoiding getting a child’s saliva in the mouth, and kissing on the lips.

## Data Availability

No datasets were generated or analysed during the current study.
